# Preparation of Coaxial-Electrospun Poly[bis(*p*-methylphenoxy)]phosphazene Nanofiber Membrane for Enzyme Immobilization

**DOI:** 10.3390/ijms131114136

**Published:** 2012-11-02

**Authors:** Shu-Gen Wang, Xin Jiang, Peng-Cheng Chen, An-Guo Yu, Xiao-Jun Huang

**Affiliations:** 1 Key Laboratory of Eco-Textile, Ministry of Education, Jiangnan University, Wuxi 214122, China; E-Mails: wshugen@yahoo.com.cn (S.-G.W.); auqfusr.1989@yahoo.com.cn (X.J.); 2 MOE Key Laboratory of Macromolecular Synthesis and Functionalization, Department of Polymer Science and Engineering, Zhejiang University, Hangzhou 310027, China; E-Mails: chenpengcheng@zju.edu.cn (P.-C.C.); yag522@zju.edu.cn (A.-G.Y.)

**Keywords:** polyphosphazene, coaxial electrospinning, stable core/sheath structure, lipase immobilization

## Abstract

A core/sheath nanofiber membrane with poly[bis(*p*-methylphenoxy)]phosphazene (PMPPh) as the sheath and easily spinnable polyacrylonitrile (PAN) as the core was prepared via a coaxial electrospinning process. Field-emission scanning electron microscopy and transmission electron microscopy were used to characterize the morphology of the nanofiber membrane. It was found that the concentration of the PAN spinning solution and the ratio of the core/sheath solution flow rates played a decisive role in the coaxial electrospinning process. In addition, the stabilized core/sheath PMPPh nanofiber membrane was investigated as a support for enzyme immobilization because of its excellent biocompatibility, high surface/volume ratio, and large porosity. Lipase from *Candida rugosa* was immobilized on the nanofiber membrane by adsorption. The properties of the immobilized lipase on the polyphosphazene nanofiber membrane were studied and compared with those of a PAN nanofiber membrane. The results showed that the adsorption capacity (20.4 ± 2.7 mg/g) and activity retention (63.7%) of the immobilized lipase on the polyphosphazene nanofiber membrane were higher than those on the PAN membrane.

## 1. Introduction

Polyphosphazenes are hybrid inorganic–organic polymers with inorganic elements in the backbone and organic side-groups [1]. The ease of structural manipulation of this kind of polymer using classical nucleophilic substitution enables the incorporation of a wide variety of side-groups. Because of this synthetic flexibility, polyphosphazenes have tunable physical and chemical properties [2–5]. These tunable physicochemical and biocompatible properties make polyphosphazenes promising candidates in different biotechnological applications, such as biosensors, controlled release, drug delivery, scaffolding materials and enzyme immobilization [6–10]. Polyphosphazenes in the form of hydrogels, films, micelles and porous particle coatings have been used in various biotechnological applications [7,11–13].

In recent decades, one-dimensional nanostructured materials have attracted much attention in the biotechnology field because of their large surface area/volume ratios [9]. Various methods have been adopted for fabricating one-dimensional nanomaterials in the form of fibers, rods, belts, tubes, spirals and rings [14]. Among these, nanofibers are special, because they are long, uniform in diameter and diverse in composition. Polymer non-woven nanofiber membranes can be simply obtained by electrospinning [15,16]. As well as a high specific surface area, nanofiber membranes possess fine porous structures, resulting in ease of accessibility and low diffusion resistance [17]. Compared with nanoparticulates, they are easily recoverable from reaction media, showing great promise for continuous operations [14]. One can envisage the combination of polyphosphazene and nanofiber morphologies to create a favorable support by uniting the high tunability and biocompatibility of polyphosphazene with the interesting characteristics of nanofiber membranes [18,19]. However, the alternating phosphorus and nitrogen atoms in the polyphosphazene backbone make the main chains flexible, usually causing the nanofiber to shrink in traditional electrospinning processes. Overcoming this disadvantage to obtain a mechanically stable polyphosphazene nanofiber membrane is a problem. Most reports have focused on molecular structural design of the polyphosphazene by introducing bulkier side-groups; other scientists have tried to blend polyphosphazene with more rigid polymers [19,20].

**Figure 1:**
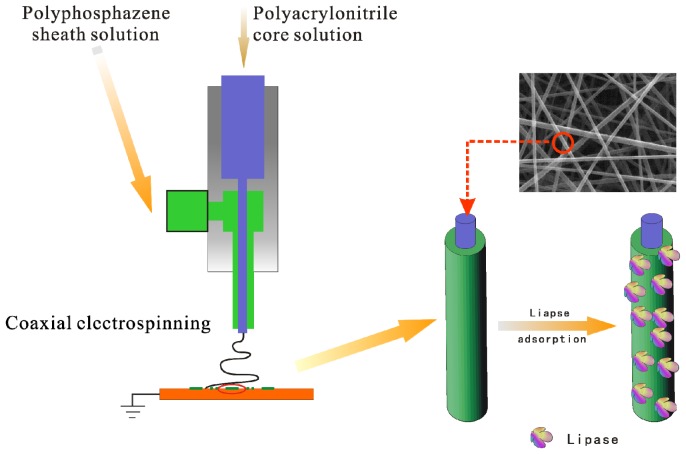
Schematic representation of the fabrication of PAN/PMPPh nanofiber membrane for lipase immobilization.

Coaxial electrospinning, which has emerged in the last decade, provides another route to overcoming this drawback. In this technique, a syringe with two compartments containing different polymer/non-polymer solutions is used to initiate a core/sheath jet, making it possible to fabricate functional composite nanostructured materials [21,22]. Coaxial electrospinning is of particular interest for materials that will not easily form fibers via electrospinning on their own [23–25]. In this study, coaxial electrospinning was used to fabricate a core/sheath nanofiber membrane with poly[bis(*p*-methylphenoxy)]phosphazene (PMPPh) as the sheath and easily spinnable polyacrylonitrile (PAN) as the core. The electrospinning conditions were systematically studied to obtain a nanofiber membrane with a stable morphology. The polyphosphazene nanofiber membrane was then used for enzyme immobilization. In our previous work, we investigated the immobilization of lipase from *Candida rugosa* on electrospun nanofiber membranes by covalent or physical adsorption, and in this study, it was again used as the model enzyme for physical attachment to the nanofiber membrane [25–28]. The process is shown schematically in Figure 1. One promising characteristic of lipases is their interfacial activation in the presence of a moderately hydrophobic interface; this can induce important conformational rearrangements and improve their activity during immobilization [14]. We deduced that the *p*-methylphenoxy side-groups can provide a moderately hydrophobic environment and enhance lipase activity during immobilization. It is expected that this work will offer a convenient pathway for flexible polyphosphazenes to form stable nanofibers for practical enzyme immobilization and biomedical applications, such as controlled release and drug delivery, as well as tissue engineering.

## 2. Results and Discussion

### 2.1. Characterization of PMPPh

The Fourier-transform infrared (FT-IR) and ^1^H nuclear magnetic resonance (NMR) analysis were conducted to exam the structure of the polyphosphazene. For these two tests, powder samples were used, and results were shown in the Supporting Information. Figure S1 shows absorption peaks at 3034 cm^−1^ and 1600 cm^−1^, characteristic of the benzene ring; the vibration peaks of CH_3_ groups bonded to the benzene ring appear at 2840–3000 cm^−1^; other characteristic absorption peaks observed at 1212 cm^−1^ and 946 cm^−1^ were ascribed to vibrations of P–O–C groups. In particular, the peaks at 1325 cm^−1^ and 823 cm^−1^ (for P–N groups), and 1192 cm^−1^ (for P=N groups) demonstrated that the polymer backbone was intact. In the ^1^H NMR spectrum in Figure S2, the peaks at 6.861 ppm and 2.17 ppm were assigned to aromatic protons and methyl protons, respectively. These results verified the success of the PMPPh synthesis, offering possibility of using this polymer for further electrospinning process. The M̄n, M̄w and polydispersity index *D* (M̄w/M̄n) of PMPPh were 7.92 × 10^5^ g/mol, 1.23 × 10^6^ g/mol and 1.55, respectively, according to gel permeation chromatography (GPC) analysis.

### 2.2. Traditional Electrospinning of PMPPh Nanofiber Membrane

**Figure 2:**
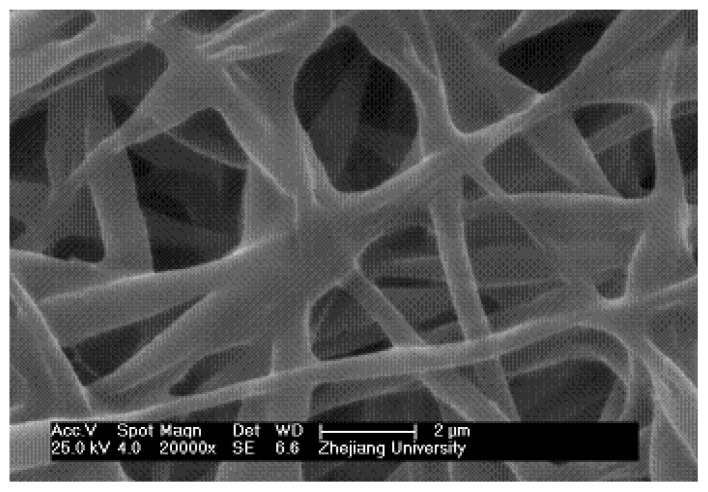
Morphology of PMPPh nanofibers obtained via traditional electrospinning.

Traditional electrospinning is a mature technique and has been widely used to fabricate polymer fibers with diameters ranging from several hundreds of micrometers down to tens of nanometers. Figure 2 shows the morphology of PMPPh nanofibers produced using this technique. In this scanning electron microscopy (SEM) image, cohesion of various degrees can be clearly observed at the intersections of different fibers; smooth, long, uniform fibers cannot be obtained. Also, the electrospun PMPPh nanofibers were difficult to detach from the collector and were prone to shrink, as seen in later observations. These phenomena were a result of the low glass-transition temperature of PMPPh (−2.91 °C). As a result, PMPPh nanofibers prepared in this way are unsuitable for practical applications.

In order to prepare PMPPh nanofibers with a stable morphology and good mechanical properties for further applications, a coaxial electrospinning technique was used to fabricate core/sheath-structured nanofibers. In these nanofibers, PAN was used as the inner support to improve the stability and mechanical strength of the nanofibers.

### 2.3. Effect of Solution Concentrations on Core/Sheath Nanofiber Morphology and Diameter

**Figure 3:**
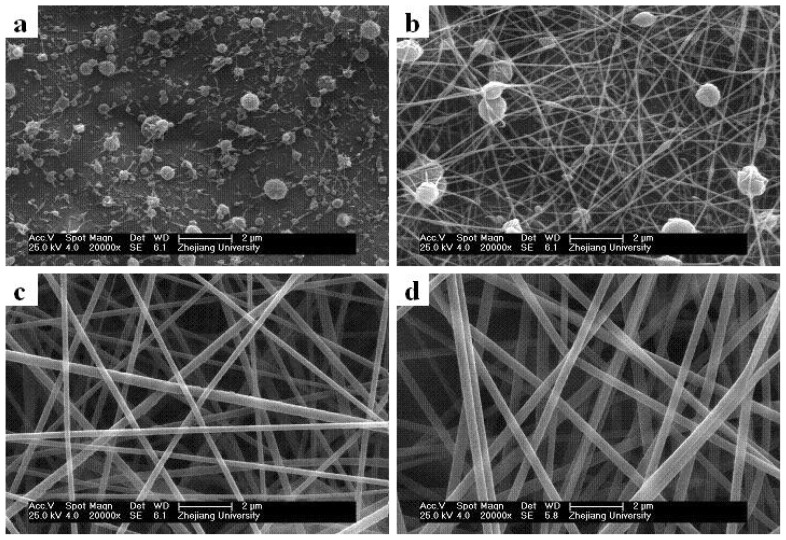
Effect of PAN concentration on the coaxial electrospinning process. PAN concentration: (**a**) 5 wt%, (**b**) 10 wt%, (**c**) 15 wt%, and (**d**) 20 wt%.

Many factors influence the diameters and morphologies of electrospun core/sheath nanofibers, and the solution properties play a significant role. Here we studied how the core (PAN) and sheath (PMPPh) solution concentrations influence the coaxial electrospinning results. The SEM micrographs in Figure 3 show the effect of PAN concentration on the fiber morphology and diameter, with the PMPPh concentration held at 15 wt%, and the flow rates for both solutions controlled to 0.2 mL/h. It can be observed that when the PAN concentration was 5 wt%, only droplets were obtained as a result of severe electrospraying. Increasing the PAN concentration to 10 wt% gave an improvement, but smooth and continuous fibers were still not obtained. Further increases in the PAN concentration resulted in continuous fibers with a uniform morphology and larger diameter of 160 ± 45 nm (Figure 3c) and 250 ± 22 nm (Figure 3d).

**Figure 4:**
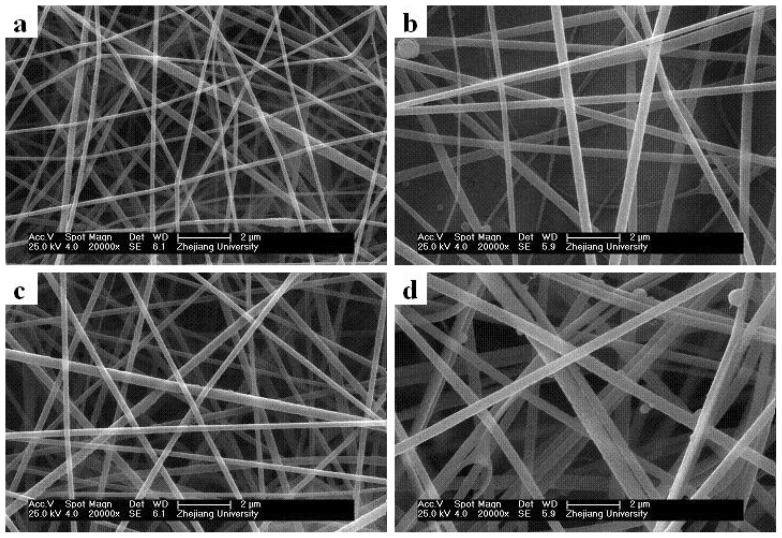
Effect of PMPPh concentration on the coaxial electrospinning process. PMPPh concentration: (**a**) 5 wt%, (**b**) 10 wt%, (**c**) 15 wt%, and (**d**) 20 wt%.

Figure 4 shows the nanofiber morphologies and diameters for different PMPPh concentrations. In these experiments, the PAN concentration was 15 wt%, with flow rates of 0.2 mL/h for the PMPPh and PAN solutions. Figure 4 shows that the average diameter of the fibers increased from 120 nm to 500 nm when the PMPPh concentration increased from 5 wt% to 20 wt%.

From the above results, it can be seen that the core solution concentration significantly influences the coaxial electrospinning process. Continuous and uniform fibers cannot be prepared if the PAN concentration is too low, and the PMPPh concentration affects the fiber diameter.

### 2.4. Effect of Core/Sheath Solution Flow Rates Ratio on Nanofiber Morphology and Diameter

**Figure 5:**
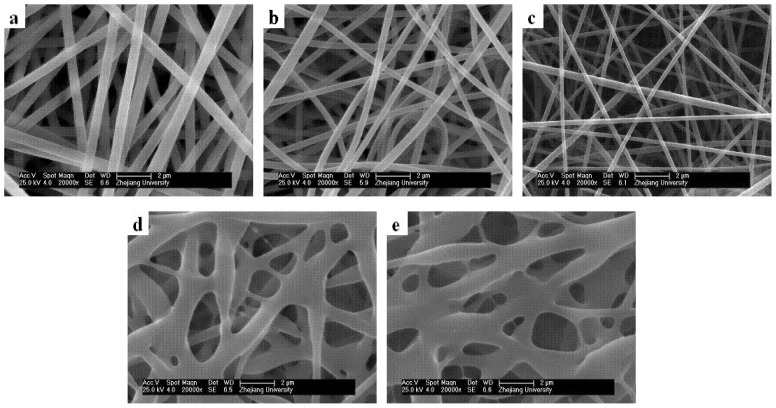
Effect of ratio of PAN solution and PMPPh solution flow rates on the coaxial electrospinning process: (**a**) 3:1, (**b**) 2:1, (**c**) 1:1, (**d**) 1:2, and (**e**) 1:3.

Unlike traditional electrospinning, coaxial electrospinning usually involves two or more spinning solutions, and the flow rates ratio strongly influences the electrospinning results. In this work, the morphologies of different fibers obtained by adjusting the ratio of the core and sheath solution flow rates were characterized using SEM, as shown in Figure 5. The concentrations of the PAN and PMPPh solutions were 15 wt%. Figure 5 shows that nanofibers with a uniform morphology were obtained when the PAN solution flow rate was higher than that of PMPPh; the average diameter of the fibers decreased from 500 ± 50 nm to 150 ± 20 nm with a decreasing core/sheath solution flow rate ratio from 3:1 to 1:1. The fiber diameter decrease was the result of the efficient elongation of the polymer solution jet in the electric field caused by the decrease in the core solution flow rate. In Figure 5d,e, serious cohesion between fibers is observed with increasing sheath solution flow rate with the core solution flow rate held constant.

**Figure 6:**
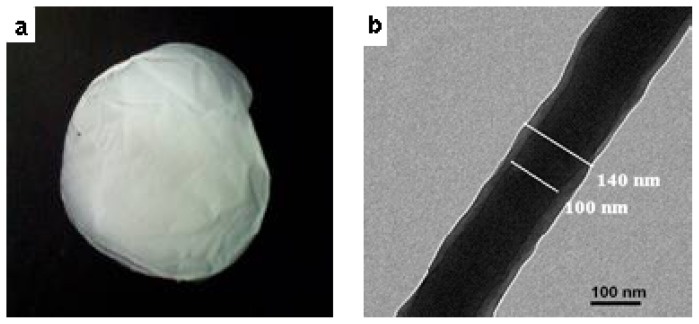
Images of coaxial-electrospun nanofiber membrane: (**a**) macroscopic image of the nanofiber membrane and (**b**) TEM image of nanofibers.

According to the discussions above, the optimized parameters for PMPPh/PAN coaxial electrospinning were 15 wt% concentrations, and 0.2 mL/h flow rates for both the PAN and PMPPh solutions. Under these conditions, the electrospun nanofiber membrane did not shrink after being detached from the collecting plate, as shown in Figure 6a. The characteristic core/sheath structure of the nanofibers was clearly confirmed by transmission electron microscopy (TEM). The overall diameter of the fibers in Figure 6b was 140 ± 10 nm, and the diameter of the core was 100 ± 10 nm. The PMPPh/PAN nanofiber membranes, as characterized in Figure 6, were used for the following enzyme immobilization tests.

### 2.5. Enzyme Immobilization on PMPPh/PAN Nanofiber Membrane

**Figure 7:**
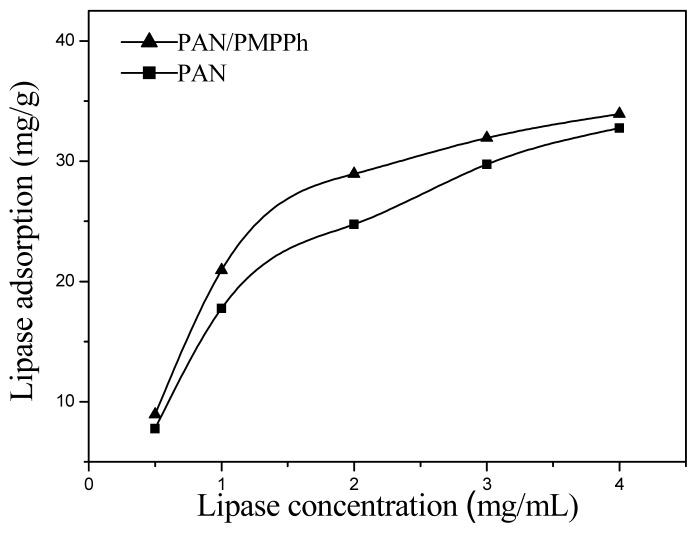
Effect of initial lipase concentration on amount of protein adsorbed on PAN (■) and PAN/PMPPh (▲) nanofiber membranes.

**Figure 8:**
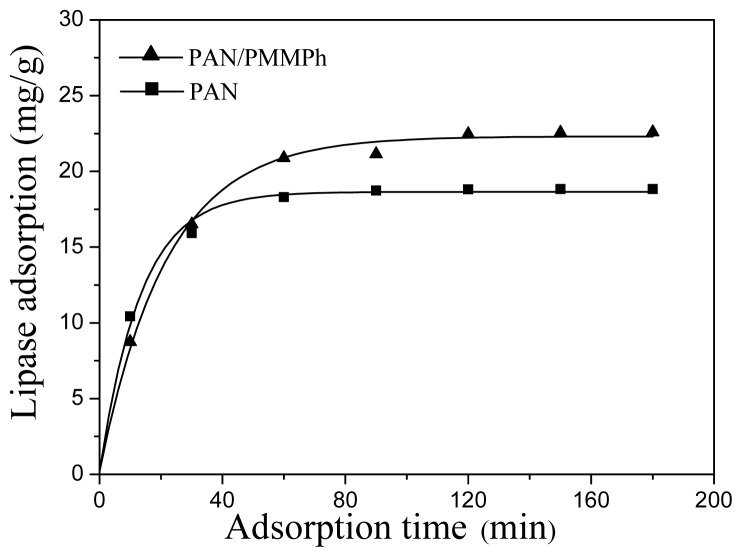
Effect of time on protein absorption on the PAN (solid) and PAN/PMPPh (dash) nanofiber membranes.

Enzyme loading reflects the extent of interaction between the enzyme and the substrate. To study the enzyme immobilization efficiency of the PMPPh/PAN nanofiber membrane, a PAN nanofiber membrane with fibers of the same diameter was used as a comparison for lipase loading measurements. Figure 7 shows the amount of lipase adsorbed on the PMPPh/PAN and PAN nanofiber membranes under different lipase concentrations. The figure shows that the enzyme loading increased with increasing lipase concentration for both supports. Furthermore, more lipase was immobilized on the PMPPh/PAN nanofiber membrane than on the PAN one. The time dependence of lipase absorption on both supports is shown in Figure 8, with the lipase concentration of 1 mg/mL used for these tests. Enzyme loading on the PMPPh/PAN nanofiber membrane reached 22.5 mg/g after absorption balance, more than the loading of 18.8 mg/g for the PAN nanofiber membrane. These values show that lipase attaches more easily to PMPPh than to PAN. As a result of the multiple conformation properties of lipase, the interfacial interaction between the support surface and the hydrophobic domain around the lipase’s active center seems to dominate the adsorption strength. Therefore, a more hydrophobic PMPPh surface can strengthen the lipase adsorption capacity on the core/sheath nanofibers.

### 2.6. Activities of Immobilized Lipases

The lipase-immobilized membrane system has its advantages. On one hand, the immobilized lipases still can catalyze a wide range of reactions such as alcoholysis, hydrolysis, trans-esterifications, aminolysis and enantiomer resolution; on the other hand, the immobilization technique offers better catalytic stability, feasible catalyst recycling, significant operation cost reduction and simplified product purification in practical applications. Thus, such system can overcome the thermal stability, reusability and recoverability limitations associated with the free enzymes, and can be widely applied in the food, pharmaceutical and detergent industries [26,29–31]. PAN is known to be a polymer with good stability and mechanical properties, thus it is widely used as supports for physical or chemical attachments of enzymes. Also, biocompatible elements and functional groups have been introduced on the supports or in the PAN backbone with the aim of improving the enzymatic activity and offering covalent binding sites for enzymes [17,32–35]. Table 1 shows some typical examples of immobilizations of lipase from *Candida rugosa* on PAN-based nanofiber membranes; *p*-nitrophenol palmitate (*p*-NPP) was used as the substrate for activity assessment. In this work, the activity retention of lipases immobilized on PAN and PMPPh/PAN nanofiber membranes were found to be 46.4% and 63.7%, respectively. The decrease in enzymatic activity after immobilization was caused by changes in the enzymatic three-dimensional structure. The increase in lipase activity on the PMPPh/PAN nanofiber membrane compared with that on the electrospun PAN nanofiber membrane can be explained by interfacial activation of the lipase: in the presence of a moderately hydrophobic surface, the lipase undergoes conformational rearrangements, yielding its “open state”, which favors the exposure of active centers, thus increasing the activity retention of the immobilized lipase [36–39].

## 3. Experimental Section

### 3.1. Materials

Polyacrylonitrile (PAN, Mη = 50,000 g/mol) was kindly supplied by Anqing Petroleum Chemical Corporation of China. Hexachlorocyclotriphosphazene (Boyuan New Material & Technology Co., Ltd. Ningbo, China) was purified by recrystallization from heptane at the temperature of 60 °C, followed by vacuum sublimation twice. Poly(dichlorophosphazene) (PDCP) was synthesized via thermally initiated ring-opening polymerization of hexachlorocyclotriphosphazene (HCCP) in a sealed Pyrex tube at 250 °C. Tetrahydrofuran (THF) was dried by refluxing over a Na/K alloy and distilled under nitrogen. Ethanol and *p*-methylphenoxy was purchased from Sinopharm Chemical Reagent Co., Ltd. (Shanghai, China). Lipase was purchased from the Huamei Biological Engineering Co., Ltd. (He’nan, China). *p*-NPP was purchased from Sigma-Aldrich Chemical Co. (St. Louis, MO, USA). All other reagents were analytical grade and used without further purification.

### 3.2. Synthesis and Analysis of PMPPh

The synthesized PDCP was rinsed with petroleum ether to remove the remaining monomers and oligomers, and then dissolved in THF. In a separate round-bottomed flask, 7.5 g of *p*-methylphenol were dissolved in 50 mL of THF, and 6 g of Na were added to the solution. After reacting for 12 h, the solution turned reddish orange. The product, methylphenolsodiumsalt solution, was then slowly added dropwise to the PDCP solution. The mixture was stirred for 48 h at room temperature. The resultant polymer was then purified by precipitation of the concentrated reaction mixture in deionized water and redissolution in THF three times, followed by precipitation in petroleum ether. The obtained elastomer was dried in a vacuum at 50 °C; the yield was 93%.

Chemical structure of PMPPh was characterized by FT-IR and ^1^H NMR. The FT-IR spectrum of PMPPh samples in KBr were obtained on a Brucker Vector 22 FT-IR spectrometer, while the ^1^H NMR spectra of sample solution in CDCl_3_ were recorded at room temperature on a Brucker Advance DMX500 nuclear magnetic resonance spectrometer. Molecular masses and molecular mass distributions were determined by GPC using THF as the solvent with a flow rate of 1.0 mL/min at 35 °C, using polymethylmethacrylate as the standard.

### 3.3. Preparation of Coaxial-Electrospun Nanofiber Membrane

To fabricate the PMPPh/PAN nanofiber membrane via a coaxial electrospinning process, PAN and PMPPh were dissolved in *N*,*N*-dimethylformamide at room temperature with gentle stirring for 12 h to form a homogeneous solution. After complete removal of air bubbles, the spinning solutions were poured into the inner and outer compartments of a coaxial plastic syringe. The electrospinning was optimized, and the required fibers were obtained using a voltage of 16 kV and a distance of 15 cm from the needle tip to the collector. A sufficiently thick fiber membrane was obtained after electrospinning for 4 h and was then dried under a vacuum at 60 °C. The electrospinning parameters for PAN and PMPPh/PAN nanofiber membranes used for lipase immobilization are shown in Table S1. The diameter and morphology of the electrospun nanofiber membrane were determined by sputter-coating with gold and examined using field-emission SEM (FE-SEM, Sirion-100, FEI, Portland, OR, USA) and TEM (JEM-1200EX, Tachikawa, Japan).

### 3.4. Immobilization of Lipase on Nanofiber Membrane

A lipase solution was prepared by dissolving an appropriate amount of lipase powder in phosphate buffer solution (PBS, 0.05 M, pH 7.0). The enzyme immobilization process was achieved by adsorption: a specific amount of nanofiber membrane was immersed in lipase solution at 25 °C for a period of time ranging from 10 min to 180 min. The lipase-adsorbed nanofiber membrane was then removed and rinsed repeatedly with PBS (0.05 M, pH 7.0) until no soluble protein was detected in the washings.

The protein concentration in the solution was determined using Coomassie Brilliant Blue reagent, following Bradford’s method [40]. Bovine Serum Albumin (BSA) was used as a standard to construct a calibration curve. The adsorption capacity of lipase on the nanofiber membrane was calculated from the protein mass balance among the initial and final lipase solutions and washings. The enzyme loading on the nanofiber membrane was defined as the amount of protein (milligrams) per gram of nanofiber membrane. Each reported value was the mean of at least three experiments, and the standard deviation was within *ca*. ±5%.

### 3.5. Assay of Lipase Activity

The activity of the immobilized lipase in an aqueous medium was determined using a previously reported method [32]. The standard conditions were a substrate solution composed of 1.0 mL of ethanol containing 14.4 mM *p*-NPP and 1.0 mL of PBS (0.05 M, pH 7.0) in an Erlenmeyer flask. The reaction was started by immersing an appropriate amount of immobilized lipase in the flask. After reaction for 5 min, 2.0 mL of sodium carbonate (0.5 M) were added to terminate the reaction, followed by centrifugation for 10 min at 10000 rpm. The supernatant (0.5 mL) was diluted 10-fold with deionized water and examined using an ultraviolet spectrophotometer at 410 nm (UV-2450, Shimadzu, Kyoto, Japan). A blank solution without added membrane was processed in the same way.

One enzyme unit is defined as the amount of biocatalyst liberating 1.0 μmol *p*-nitrophenol min^−1^ in this condition. Specific activity was the number of lipase units per milligram of protein. Activity retention of immobilized enzyme was defined as the ratio of specific activity of immobilized enzyme to that of a free one.

## 4. Conclusions

In this work, a coaxial electrospinning technique was used to fabricate PMPPh/PAN nanofiber membranes with a stable morphology; the parameters affecting the electrospinning process were studied systematically. Under the optimized electrospinning conditions of a 15 wt% concentration and 0.2 mL/h flow rate for both PMPPh and PAN, nanofibers of diameter 150 ± 20 nm and a clear core/sheath structure were obtained. The electrospun PMPPh/PAN coaxial nanofiber membrane was studied with respect to lipase immobilization. Because of the moderately hydrophobic and biocompatible surface of the membrane, the immobilized lipase on the PMPPh/PAN coaxial nanofiber had an absorption capacity of 20.4 ± 2.7 mg/g and an activity retention as high as 63.7%.

## Supplementary Information



## Figures and Tables

**Table 1:** Performance of lipase from Candida rugosa immobilized on various PAN-based nanofiber membranes.

Support	Immobilization method	Fiber diameter (nm)	Enzyme loading (mg/g fiber)	Activity retention (%)	References
PAN NF a	Physical	150 ± 20	18.8 ± 2.4	46.4	This work
PMPPh/PAN NF b	Physical	150 ± 20	20.4 ± 2.7	63.7	This work
PANCMPC NF c	Physical	90 ± 20	22.2 ± 1.7	72.9 ± 0.8	[33]
PANCHEMA d	Chemical	80–50	16.2 ± 1.1	40.6 ± 0.6	[17]
PANCMA NF e	Chemical	100	21.2 ± 0.71	37.6 ± 1.8	[32]
Chitosan-tethered PANCMA NF	Chemical	NA f	22.5 ± 0.75	45.6 ± 1.8	[34]
Gelatin-tethered PANCMA NF	Chemical	NA	20.7 ± 0.75	49.7 ± 1.8	[34]
PAN NF	Chemical	150–300	21.2 ± 1.3	81.3 ± 1.1	[35]

a Polyacrylonitrile nanofiber;

b Core-sheath poly[bis(*p*-methylphenoxy)]-phosphazene/polyacrylonitrile nanofiber;

c Poly[acrylonitrile-*co*-(2-methacryloyloxyethyl phosphoryl-choline)] nanofiber;

d Poly(acrylonitrile-*co*-2-hydroxyethyl methacrylate) nanofiber;

e Poly(acrylonitrile-*co*-maleic acid) nanofiber;

f Not available.
