# Supplementary material for: Preparation of Coaxial-Electrospun Poly[bis(p-methylphenoxy)]phosphazene Nanofiber Membrane for Enzyme Immobilization
Source: Int J Mol Sci. 2012 Nov 2;13(11):14136–48. doi: 10.3390/ijms131114136 (PMC3509571; doi:10.3390/ijms131114136)
Supplement: Supplementary file 1 [file ijms-13-14136-s001.pdf]

Supplementary Information

Table S1. Electrospinning parameters for PAN and PMPPh/PAN nanofiber membranes used for lipase immobilization.

Material	Voltage (kV)	Collecting distance (cm)	Flow rate (mL/h)	Temperature (°C)	Humidity
PAN	16	15	0.4	25	60%
PMPPh/PAN	16	15	0.2 for both solutions	25	60%

Figure S1. FT-IR spectrum of PMPPh.

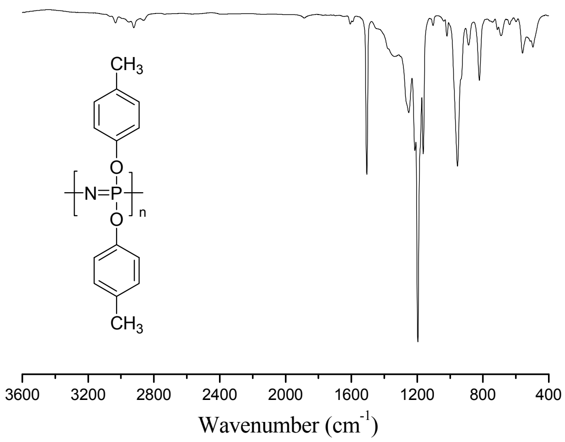

Figure S2. ^{1}H NMR spectrum of PMPPh.

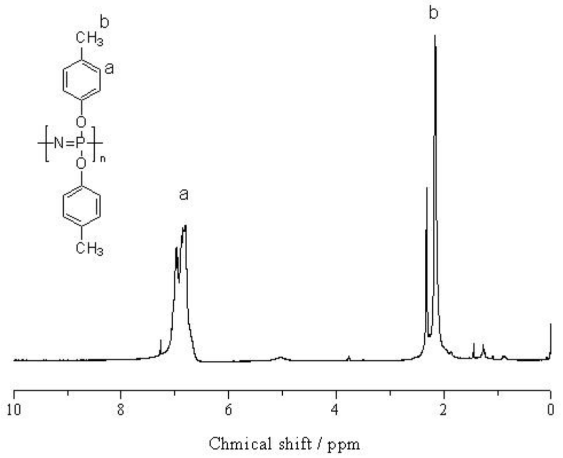

© 2012 by the authors; licensee MDPI, Basel, Switzerland. This article is an open access article distributed under the terms and conditions of the Creative Commons Attribution license (http://creativecommons.org/licenses/by/3.0/).
